# Incidence and risk factors of progressive nasal inner nuclear layer thickening after surgical peeling of epiretinal membrane

**DOI:** 10.1038/s41598-022-11609-7

**Published:** 2022-05-10

**Authors:** Hyun Young Park, Jong Yun Yang, Hyo Song Park, Hyesun Kim

**Affiliations:** 1 Siloam Eye Hospital, #181 Deungchon-ro, Gangseo-gu, Seoul, 07668 Republic of Korea; 2 Department of Ophthalmology, College of Medicine, The Institute of Vision Research, Yonsei University, Seoul, Republic of Korea; 3 Eyejun Ophthalmic Clinic, Seoul, Republic of Korea

**Keywords:** Retinal diseases, Vision disorders, Predictive markers

## Abstract

To assess incidence and risk factors of postoperative progressive nasal inner nuclear layer (INL) thickening after epiretinal membrane (ERM) surgery. Progressive nasal INL thickening was defined as 1.5-fold increase in thickness of nasal INL after ERM surgery compared to preoperative examination. Kaplan–Meier survival analysis was done to compare the cumulative risk ratio between groups stratified by presence of progressive nasal INL thickening. Logistic regression was performed to identify possible risk factors. Progressive nasal INL thickening occurred in 13.0% of ERM removal patients. Patients without progressive nasal INL thickening showed better visual acuity recovery compared to patients with nasal INL thickening (*p* = 0.029). Presence of cystoid space in inner retinal layer before surgery (odds ratio [OR] = 0.143, 95% confidence interval [CI] 0.028–0.736; *p* = 0.020), older age (OR = 0.896, 95% CI 0.817–0.982, *p* = 0.020), and thicker preoperative central macular thickness (OR = 0.994, 95% CI 0.988–1.000, *p* = 0.039) were correlated inversely with thickening of nasal INL. Correlation between nasal INL thickness and postoperative visual outcome was significant. Absence of cystoid space before ERM surgery, younger age, and thinner central macular thickness were risk factors for progressive postoperative nasal INL thickening. Progressive nasal INL thickening may serve as a new biomarker for worsened visual symptom after ERM surgery.

## Introduction

Epiretinal membrane (ERM) is a common macular disease characterized by fibrocellular tissue which grows on inner surface of retina^1–3^. Decreased vision and metamorphopsia are major symptoms of patients with ERM^4,5^. Surgical removal of membrane is performed when symptom aggravation or worsening of structural distortion exists^6,7^. However, even after successful removal of membrane, functional outcomes vary, and some patients even complain of worsened symptoms.

Studies have investigated relationship between structural changes of inner retinal layer and functional outcome after ERM surgery^5,7–9^. Lately, change in thickness of inner nuclear layer (INL) following ERM surgery is emerging as a new biomarker for foreseeing degree of metamorphopsia. Studies with spectral domain-optical coherence tomography (OCT) revealed that INL thickness is related to visual acuity and metamorphopsia to tangential retinal displacement in ERM patients^3,10–14^.

Recently, some studies quantitatively analyzed inner retinal layer in each subfield after ERM surgery and asymmetric change of inner retinal layer thickness regarding retinal subfield was suggested^15,16^. In a study with Early Treatment Diabetic Retinopathy Study (ETDRS) grid, it was reported that thickness of nasal parafovea decreased insufficiently after ERM surgery compared to other subfields^11,17^. However, there are no known studies evaluating the correlation between asymmetric changes of inner retinal layer and functional outcome after ERM surgery. In a series of such patients with worsening symptoms even after ERM surgery, we clinically experienced progressive thickening of inner retinal layer at nasal macula, especially INL, and shortened of distance between fovea and optic disc.

The purpose of this study is to retrospectively assess incidence and onset of progressive nasal INL thickening after ERM surgery and to identify associated risk factors. Relationship between post-ERM surgery functional outcome and nasal INL thickening was analyzed.

## Results

Among 304 eyes of 296 patients who underwent ERM surgery, 108 eyes of 107 patients were enrolled. 28 eyes met one of the exclusion criteria for possible cause of secondary ERM (14 with proliferative diabetic retinopathy, 6 with retinal vascular diseases, 3 with uveitis, 3 with central serous chorioretinopathy, 1 with retinal detachment and 1 with retinoschisis) and 166 eyes did not have OCT images which met inclusion criteria (153 with inappropriate protocol; OCT images with less than 25 line B-scan so which were unable to analyze within a 3-mm diameter circle, 11 without postoperative OCT images, 2 with insufficient quality of OCT images for analysis). One eye with postoperative endophthalmitis and one eye with macular hole after ERM surgery were also excluded.

Patient demographics were as follows. Mean age was 69.7 years (range 37–85). Mean observation period after surgery was 251 days (range 22–1169). 71 patients were female (65.7%). 59 eyes (54.6%) underwent surgery on left side. 51 patients (47.7%) had hypertension and 23 patients (21.5%) had diabetes without sign of either diabetic retinopathy or diabetic macular edema. The baseline patient characteristics are listed in Table 1.

**Table 1:** Baseline patient characteristics.

Variables^a^	Number
Gender (male:female)	37 (34.3%):71 (65.7%)
Age (years)	69.7 ± 8.5 (37–85)
Laterality (right:left)	49 (45.4%):59 (54.6%)
Anesthesia (retrobulbar:general)	105 (97.2%):3 (2.8%)
Initial BCVA (logMAR)	0.55 ± 0.25 (0.1–1.0)
Follow-up duration (days)	251 ± 229 (22–1169)
**Intraoperative procedure**	
Combined cataract surgery	84 (78.5%)
ILM peeling	93 (86.9%)
Fluid-air exchange	5 (4.7%)
**Underlying disease**	
HTN	51 (47.2%)
DM	23 (21.3%)

*SD* standard deviation, *logMAR* logarithm of the minimum angle of resolution, *BCVA* best corrected visual acuity, *ILM* internal limiting membrane, *HTN* hypertension, *DM* diabetes mellitus.

^a^Eyes of a patient who received epiretinal membrane surgery on both eyes were counted separately as twice in the baseline demographics above.

### Progressive nasal inner nuclear layer thickening

Among 108 eyes that underwent ERM surgery, 14 eyes (13.0%) exhibited progressive nasal INL thickening as revealed by OCT segmentation analysis. Comparison of characteristics of ERM patients with and without progressive nasal INL thickening is listed in Table 2. Mean duration of onset was 73.71 ± 125.71 days (range: 22–514) and after excluding two outliers (114 days and 514 days respectively), mean duration of onset was 31.17 ± 7.80 days (range: 22–49).

**Table 2:** Demographics and clinical characteristics of epiretinal membrane patients with and without progressive nasal inner nuclear layer thickening after the surgery.

Variables	With INL thickening	Without INL thickening	*p*
Eyes (n)	14	94	
Age (years)	63.64 ± 11.80	70.55 ± 7.69	**0.004**
Male:female	4:10	33:61	0.631
HTN (%)	8 (57.1%)	43 (45.7%)	0.425
DM (%)	3 (21.4%)	20 (21.3%)	0.990
Preoperative BCVA (logMAR)	0.22 ± 0.27	0.33 ± 0.25	0.128
BCVA (logMAR) at 1 month after the surgery	0.19 ± 0.24	0.18 ± 0.23	0.892
Postoperative BCVA (logMAR)	0.17 ± 0.27	0.15 ± 0.20	0.780
Change in BCVA (logMAR) at 1 month after the surgery	− 0.03 ± 0.19	− 0.15 ± 0.24	0.077
Change in BCVA (logMAR) at final visit	− 0.05 ± 0.21	− 0.18 ± 0.20	**0.029**
Combined cataract surgery (%)	13 (92.9%)	71 (75.5%)	0.146
ILM peeling (%)	13 (92.9%)	80 (85.1%)	0.434
Fluid-air-exchange (%)	0 (0.0%)	5 (5.3%)	0.377
Follow-up duration (days)	237.79 ± 198.13	235.26 ± 235.05	0.815
Presence of IS/OS disruption at initial presentation (%)	2 (14.3%)	23(24.5%)	0.399
Presence of cystoid space at initial presentation (%)	2 (14.3%)	53 (56.4%)	**0.003**
Preoperative central macular thickness (μm)	351.14 ± 93.34	416.11 ± 117.33	0.051
Preoperative average fovea thickness (μm)	386.14 ± 72.36	436.53 ± 87.80	**0.043**
Change in distance between disc and fovea (μm)	− 166.43 ± 177.12	− 111.85 ± 222.42	0.383

*INL* inner nuclear layer, *HTN* hypertension, *DM* diabetes mellitus, *BCVA* best corrected visual acuity, *logMAR* logarithm of the minimum angle of resolution, *ILM* internal limiting membrane, *CMT* central macular thickness, *IS/OS* inner and outer segment.

Significant values are in [bold].

Preoperative mean best corrected visual acuity (BCVA) in progressive nasal INL thickening group was 0.22 ± 0.27 logarithm of the minimum angle of resolution (logMAR) units and it improved to 0.17 ± 0.27 logMAR units at final follow-up. Preoperative mean BCVA in group without nasal INL thickening was 0.33 ± 0.25 logMAR units and mean BCVA at final follow-up was 0.15 ± 0.20 logMAR units, showing improvement in visual acuity. There was no statistical difference between two groups before and after the surgery (*p* = 0.128 and 0.780 respectively). Patients with nasal INL thickening had − 0.05 ± 0.21 logMAR units of BCVA improvement, while patients without nasal INL thickening had − 0.18 ± 0.20 logMAR units of improvement after ERM surgery (Table 2, *p* = 0.029).

### Risk factors

**Figure 1:**
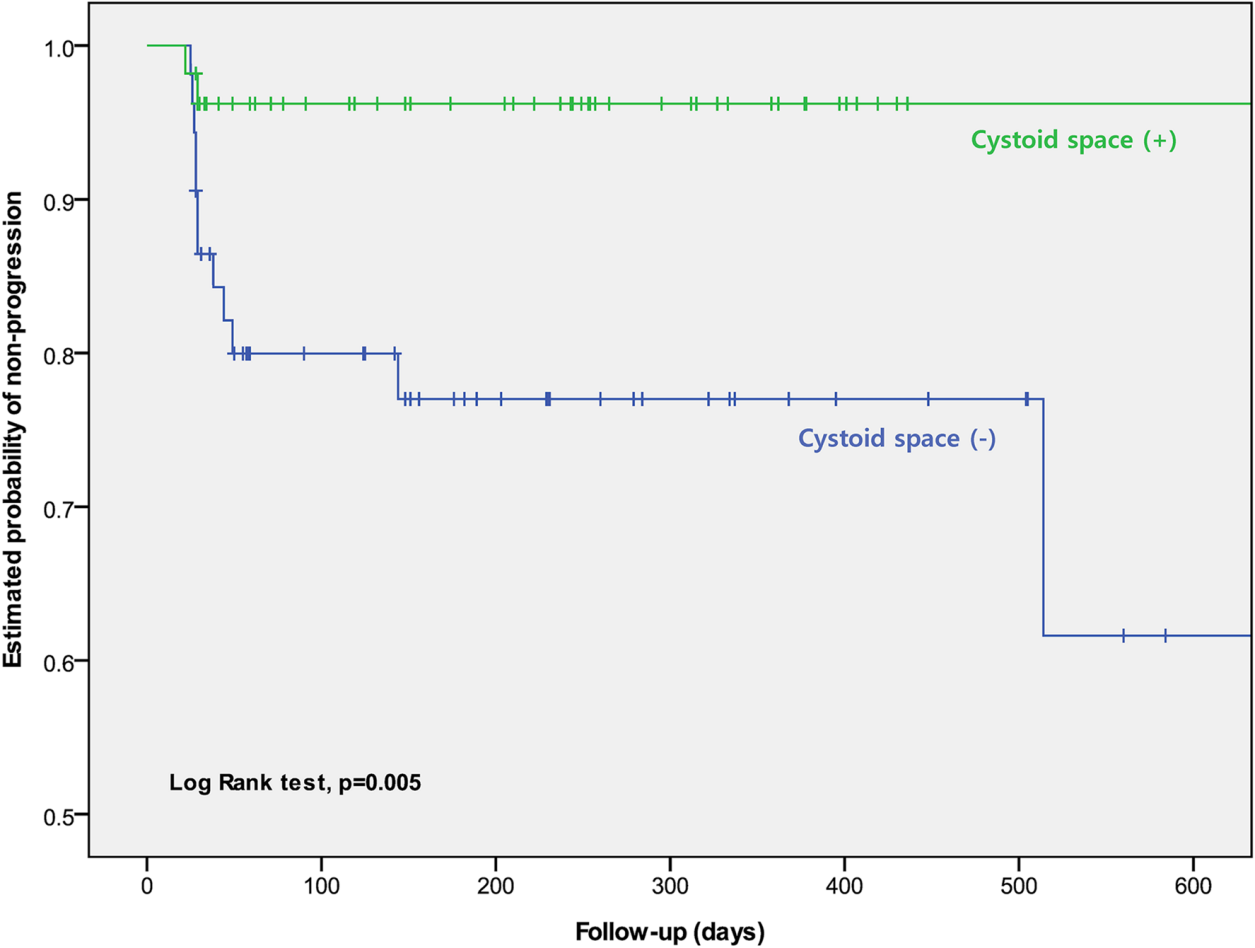
Kaplan–Meier analysis of the probability to remain without progressive nasal inner nuclear layer thickening in epiretinal membrane patients after the surgery. Log-rank test comparing eyes with or without cystoid space showed statifically significant differences.

Kaplan–Meier analysis was performed between groups stratified by presence of cystoid space before surgery. Eyes without cystoid space before surgery had significant cumulative probability of nasal INL thickening on Kaplan–Meier analysis (Fig. 1, Log-rank test, *p* = 0.005). Logistic regression analyses were done to identify possible factors associated with progressive nasal INL thickening in ERM removal surgery. Presence of cystoid space before surgery, older age, and thicker average foveal thickness were strongly associated with progressive nasal INL thickening inversely in univariate analysis (Table 3, *p* = 0.010, 0.010, 0.048 respectively). To figure out whether these variables were independently associated with progressive nasal INL thickening, multivariate analysis was performed with the variables yielding *p*-values < 0.20. Presence of cystoid space before surgery was revealed as the strongest protective factor against nasal INL thickening (odds ratio [OR] = 0.143, 95% confidence interval [CI] 0.028–0.736, *p* = 0.020). Older age (OR = 0.896, 95% CI 0.817–0.982, *p* = 0.020) and thicker preoperative central macular thickness (OR = 0.994, 95% CI 0.988–1.000, *p* = 0.039) were also significantly associated as protective factors against nasal INL thickening (Table 4).

**Table 3:** Univariate logistic regression analysis of nasal inner nuclear layer thickening in epiretinal membrane patients after surgery.

Variable	Univariate logistic regression	
	β (95% CI)	*p*
Age (years)	0.922 (0.867–0.981)	**0.010**
Gender (female)	0.739 (0.2158–2.541)	0.632
HTN	1.581 (0.509–4.913)	0.428
DM	1.009 (0.257–3.966)	0.990
Preoperative BCVA (logMAR)	0.105 (0.006–2.007)	0.135
Anesthesia (Retrobulbar)	3.538 (0.299–41.821)	0.316
Laterality (Right)	1.124 (0.362–3.493)	0.840
Combined cataract surgery	4.211 (0.522–33.966)	0.177
ILM peeling	2.275 (0.275–18.797)	0.446
Presence of IS/OS disruption at initial presentation	1.944 (0.405–9.334)	0.406
Presence of cystoid space at initial presentation	0.129 (0.027–0.608)	**0.010**
Preoperative central macular thickness (μm)	0.995 (0.990–1.000)	0.055
Preoperative average fovea thickness (μm)	0.993 (00.987–1.000)	**0.048**

*HTN* hypertension, *DM* diabetes mellitus, *BCVA* best corrected visual acuity, *logMAR* logarithm of the minimum angle of resolution, *ILM* internal limiting membrane, *IS/OS* inner and outer segment.

Significant values are in [bold].

**Table 4:** Multivariate logistic regression analysis of nasal inner nuclear layer thickening in epiretinal membrane patients after the surgery.

Variable	Multivariable logistic regression	
	β (95% CI)	*p*
Age (years)	0.896 (0.817–0.982)	**0.020**
Preoperative BCVA (logMAR)	0.595 (0.021–16.800)	0.761
Combined cataract surgery	21.637 (0.582–804.598)	0.096
Presence of cystoid space before surgery	0.143 (0.028–0.736)	**0.020**
Preoperative central macular thickness (μm)	0.994 (0.988–1.000)	**0.039**
Preoperative average foveal thickness (μm)	0.997 (0.985–1.010)	0.693

*BCVA* best corrected visual acuity, *logMAR* logarithm of the minimum angle of resolution.

Significant values are in [bold].

**Figure 2:**
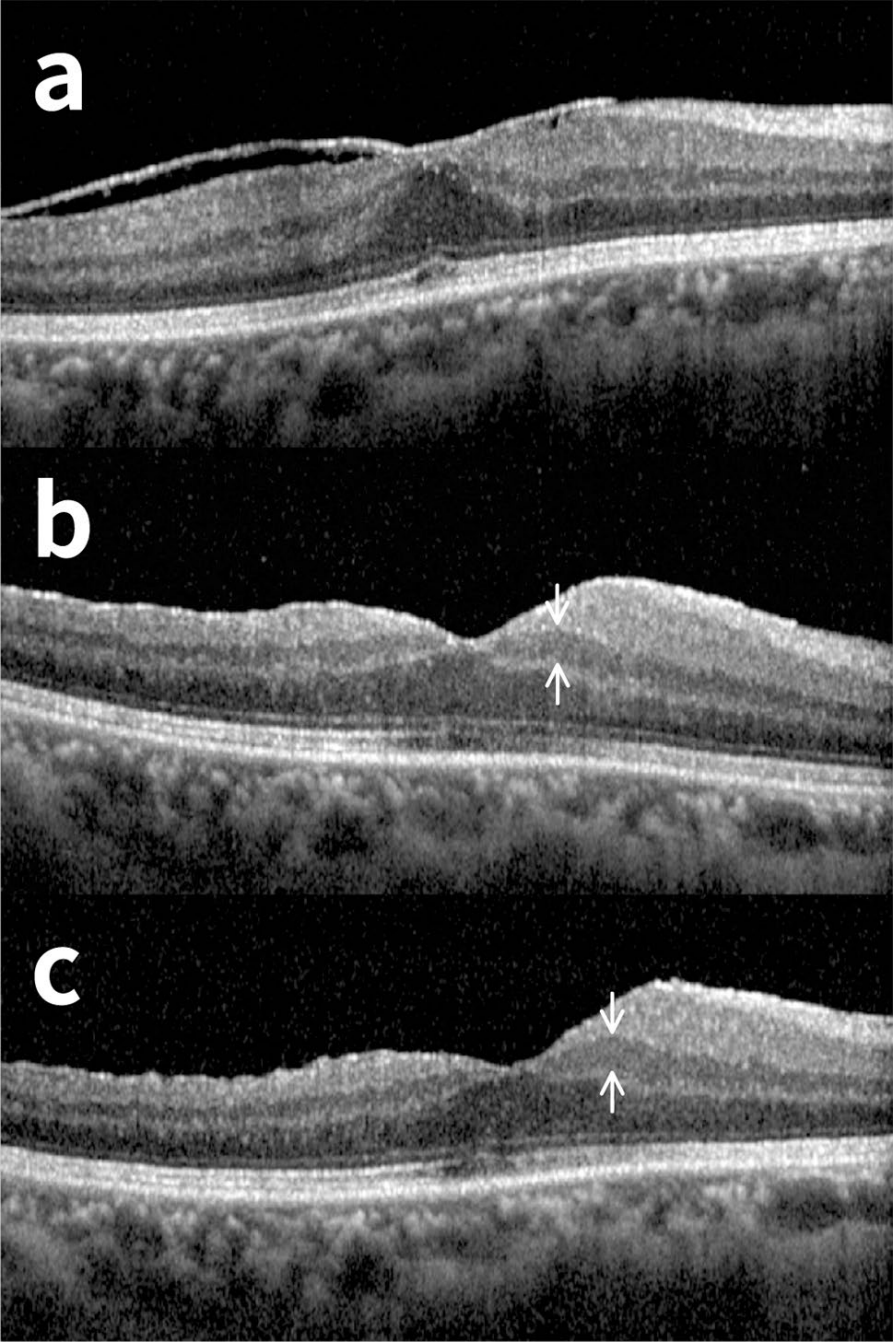
A representative case of nasal inner nuclear layer (INL) thickening patient after epiretinal membrane (ERM) surgery. A 48-year-old female with ERM had metamorphopsia and she underwent surgical peeling of ERM on her right eye. Combined cataract surgery and internal limiting membrane peeling were performed together. (**a**) Preoperative optical coherence tomography (OCT) image. (**b**) OCT images taken 38 days after the surgery. The thickness of nasal INL had increased after the surgery. (**c**) At last visit (580 days after the surgery), the nasal INL had remained thickened, best corrected visual acuity remained the same (20/25 for both preoperatively and postoperatively), and subjective metamorphopsia had not been improved. The distance between fovea and disc decreased after the removal of ERM.

**Figure 3:**
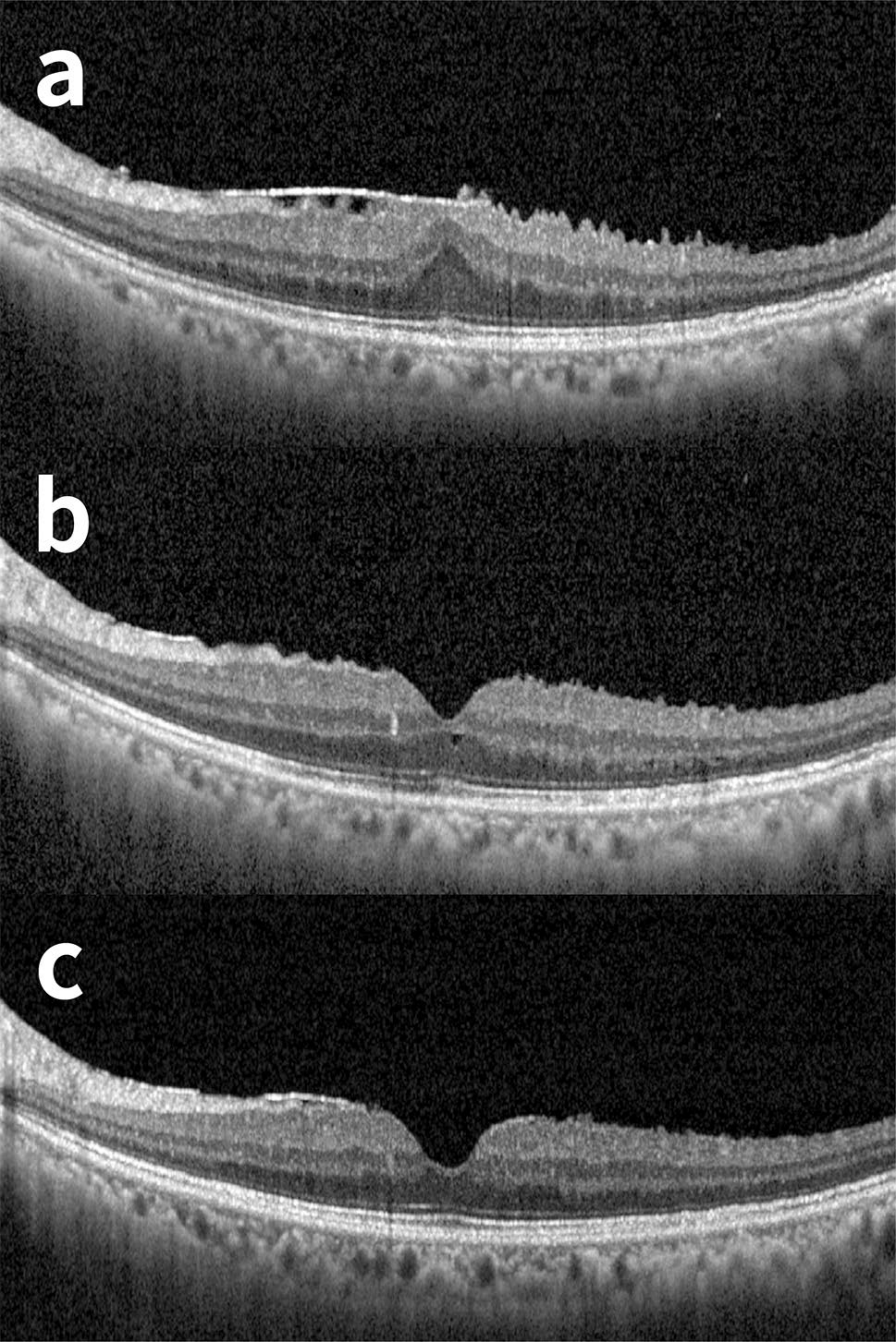
A representative case of non-nasal inner nuclear layer (INL) thickening after epiretinal membrane (ERM) surgery. A 66-year old male with hypertension and hepatitis B visited our clinic for macropsia on his left eye and ERM was revealed on fundus exam. Combined cataract surgery and peeling of ERM were performed under retrobulbar anesthesia. Internal limiting membrane was not peeled off. (**a**) Preoperative optical coherence tomography (OCT) image. At initial, the best corrected visual acuity (BCVA) was 20/32. (**b**) OCT images taken 31 days after the surgery. BCVA has been improved to 20/20. (**c**) At last visit (230 days after the surgery), the structural deformity was not present and BCVA was 20/20 and subjective macropsia has been improved.

Representative cases of nasal INL thickening group and non-nasal INL thickening group are shown in Figs. 2 and 3, respectively. A 48-year-old woman presented with metamorphopsia and ERM was diagnosed on fundus exam and OCT imaging. Surgical peeling of ERM combined with cataract surgery, and internal limiting membrane (ILM) peeling were performed. At initial visit before surgery (Fig. 2a), her BCVA was 20/25. 38 days after the surgery, her BCVA was 20/30, and progressive nasal INL thickening was revealed on OCT imaging for the first time (Fig. 2b). At her last visit (580 days after the surgery), her BCVA was 20/25, nasal INL became thicker, and she still had metamorphopsia (Fig. 2c). In the other case, a 66-year-old male with ERM presented with macropsia. Combined with cataract surgery, surgical peeling of ERM without ILM peeling was performed. At initial visit (Fig. 3a), his BCVA was 20/32. 31 days after the surgery, his BCVA had improved to 20/20 without structural deformity at nasal INL area (Fig. 3b). At the last visit (230 days after the surgery), his BCVA remained 20/20 and no definite thickness change was present at nasal INL area (Fig. 3c).

## Discussion

In this study, we found out that nasal INL thickness increased after the ERM surgery in 13.0% of patients. In nasal INL thickening group, mean improvement of BCVA was by 0.05 logMAR units, whereas mean improvement of BCVA was by 0.18 logMAR units in non-nasal INL thickening group. Previous studies reported that visual acuity improved within 10 ETDRS letters after ERM surgery^18,19^. Our outcomes only showed comparable improvement of BCVA in non-nasal INL thickening group. We confirmed that successful surgical ERM peeling resulted in sufficient visual improvement in non-nasal INL thickening group, but not in nasal INL thickening group. This reveals strong inverse correlation between thickening of nasal INL and degree of functional improvement after ERM surgery.

Previous studies have reported relationship between retinal microstructure and functional visual outcome (such as visual acuity and degree of metamorphopsia) in ERM patients^11,13,20,21^. Especially, structural deformity in INL is known to be significantly related to visual acuity and degree of metamorphopsia^20,21^. Zou et al.^21^ revealed that preoperative INL thickness in foveal, parafoveal, and perifoveal region correlates with postoperative improvement of visual acuity in 35 ERM patients. Okamoto et al.^13^ reported that INL thickness had significant correlation with degree of metamorphopsia at each time point in ERM patients after the surgery. Ichikawa et al.^11^ measured INL thickness on each retinal quadrant and verified that INL thickness is significantly correlated with tangential retinal displacement and degree of metamorphopsia. In this study, we revealed that patients with non-progressive nasal INL thickening showed better visual acuity gain after ERM surgery, and this finding suggests that progressive thickening of nasal INL is correlated with worse functional outcome.

We found that absence of cystoid space before ERM surgery, younger age, and thinner central macular thickness were risk factors for progressive nasal INL thickening after ERM surgery. Especially, presence of cystoid space was revealed as the strongest protective factor. Presence of the cystoid space itself indicates loss of retinal parenchymal cell, suggesting decreased cell density in foveal area. Surgical peeling of the membrane may have reduced mechanical traction on retina, resulting in cellular migration to fill empty space. Also, due to decreased cell density in retinal layer, change of thickness might have been relatively small. Older age and thicker central macular thickness might also indicate decreased cell density, allowing more active cellular migration after surgery.

There has been limited number of studies focused on nasal macular area. Ichikawa et al.^11^ had revealed that mean nasal INL thickness increased 1 month after the surgery, and then decreased, without statistically significant difference compared to the initial presentation. Treumer et al.^17^ suggested that retinal thickness decreased insufficiently in nasal and central area of macula after the ERM surgery in 33 eyes, compared to 30 normal eyes without ERM. These studies suggested possibility of asymmetric inner retinal layer thickness change after ERM surgery, but they did not include time course of change in nasal INL thickness. In our study, nasal INL thickness was increased steadily in nasal INL thickening group. On the other hands, nasal INL thickness maintained steady in non-nasal INL thickening group, which was consistent with the finding of previous report^17^. Also, the improvement of BCVA was significant in non-nasal INL thickening group, whereas that was insufficient in nasal INL thickening group. Thus, it is necessary to monitor closely on changes in nasal INL thickness in order to predict functional prognosis of patients.

Then there remains one question: what is so special with the nasal macular area? One possible hypothesis is that nasal dragging of macula might have occurred after the surgery. Datlinger et al.^22^ proposed that fovea moves toward optic disc within the first postoperative day of ERM surgery and that distance between fovea and optic disc is significantly related to initial BCVA and central macular thickness. They suggested that surgical removal of ERM induces iatrogenic force on retina, especially on Muller cells and photoreceptors, resulting in foveal migration toward optic disc. In our study, the distance between fovea and disc has decreased in both groups stratified by progressive nasal INL thickening (Table 2). Although there was no statistically significant difference between two groups, the degree of reduction was relatively larger in nasal INL thickening group. This supports the argument that reduction in distance between fovea and optic disc is associated with visual outcome, which is consistence with their hypothesis.

There are some limitations in this study. Firstly, due to retrospective nature of the study, we could not evaluate the quantitative degree of metamorphopsia before and after the surgery. BCVA alone has limitations in reflecting visual discomfort. Further studies might be needed to investigate the relationship between postoperative nasal INL change and visual parameters such as metamorphopsia, stereopsis, or contrast sensitivity. Secondly, the distance between disc and fovea was measured manually. To reduce errors with manual measurement, imaging analysis was done by two independent authors and final confirmation was done by two retinal specialists. Furthermore, because cataract surgery was performed concurrently with ERM removal in most cases, we could not analyze effect of cataract surgery in this study. Finally, criterion for evaluation of progressive nasal INL thickening—which was defined as 1.5-fold increase of nasal INL thickness—was determined rather subjectively by authors.

In conclusion, we confirmed that thickness of nasal INL increases in 13.0% of patients after ERM surgery, and that visual outcome is significantly correlated with postoperative nasal INL thickening. This morphologic change occurs mostly within one month after surgery, and therefore monitoring microstructure in this period is critical for evaluation of visual outcome. Presence of cystoid space at initial presentation, older age, and thicker central macular thickness may act as protective factors against progressive nasal INL thickening. Further studies should focus on mechanism of nasal INL thickening and foveal dragging to disc, and on relationship of nasal INL thickening and metamorphopsia score and other measures of visual function such as visual field index.

## Methods

This study was conducted as a retrospective medical record review of patients with diagnosis of ERM who underwent pars plana vitrectomy between March 2017 and April 2020 at Siloam Eye Hospital. This study was approved by Institutional Review Board of Siloam Eye Hospital (approval number 2021-1) and was conducted in accordance with tenets of the Declaration of Helsinki. The requirement of informed consent was waived by Institutional Review Board of Siloam Eye Hospital due to the study’s retrospective design.

### Subjects and surgical procedure

Patients who had (1) confirmed ERM on dilated fundus exam and on OCT images and (2) undergone surgical ERM peeling were selected. Eyes with missing OCT images before or after surgery, eyes with OCT images do not satisfy macular volume scan protocol as described below, or eyes with OCT images of insufficient image quality resulting in segmentation failure were excluded. Eyes with history of retinal disorders other than ERM such as diabetic retinopathy, age-related maculopathy, previous retinal detachment, macular hole, and uveitis were excluded. Eyes with previous intraocular surgery other than cataract surgery were also excluded. Eyes with postoperative complications which required treatment or re-operation were excluded. Every patient’s medical history and preoperative ophthalmic data were reviewed. BCVA was recorded using Snellen visual acuity charts. Visual acuity measurement was converted to LogMAR for statistical analysis. On each visit, fundus photography, autofluorescence, OCT scans were taken.

All surgical procedures were performed under retrobulbar or general anesthesia by 5 retinal specialists. When advanced cataract was present, with lens opacity of C2, N2 or greater according to the Lens Opacities Classification System (LOCS) III, phacoemulsification and intraocular lens implantation was performed with vitrectomy. Standard three-port 23-gauge or 25-gauge pars plana vitrectomy (according to surgeon’s preferences) was performed. After core vitrectomy, complete posterior vitreous detachment was induced. If the ridge of the ERM was visible, membrane peeling was started from the edge by applying tangential traction. When the edge was not identified, microvitreoretinal blade was used to create the edge. Epiretinal membrane peeling was performed including 3 disc diameters area around fovea. Additional ILM peeling with aid of indocyanine green solution or air-fluid exchange was performed if necessary. All of the surgeons followed the procedure described above.

### Imaging and image analysis

**Figure 4:**
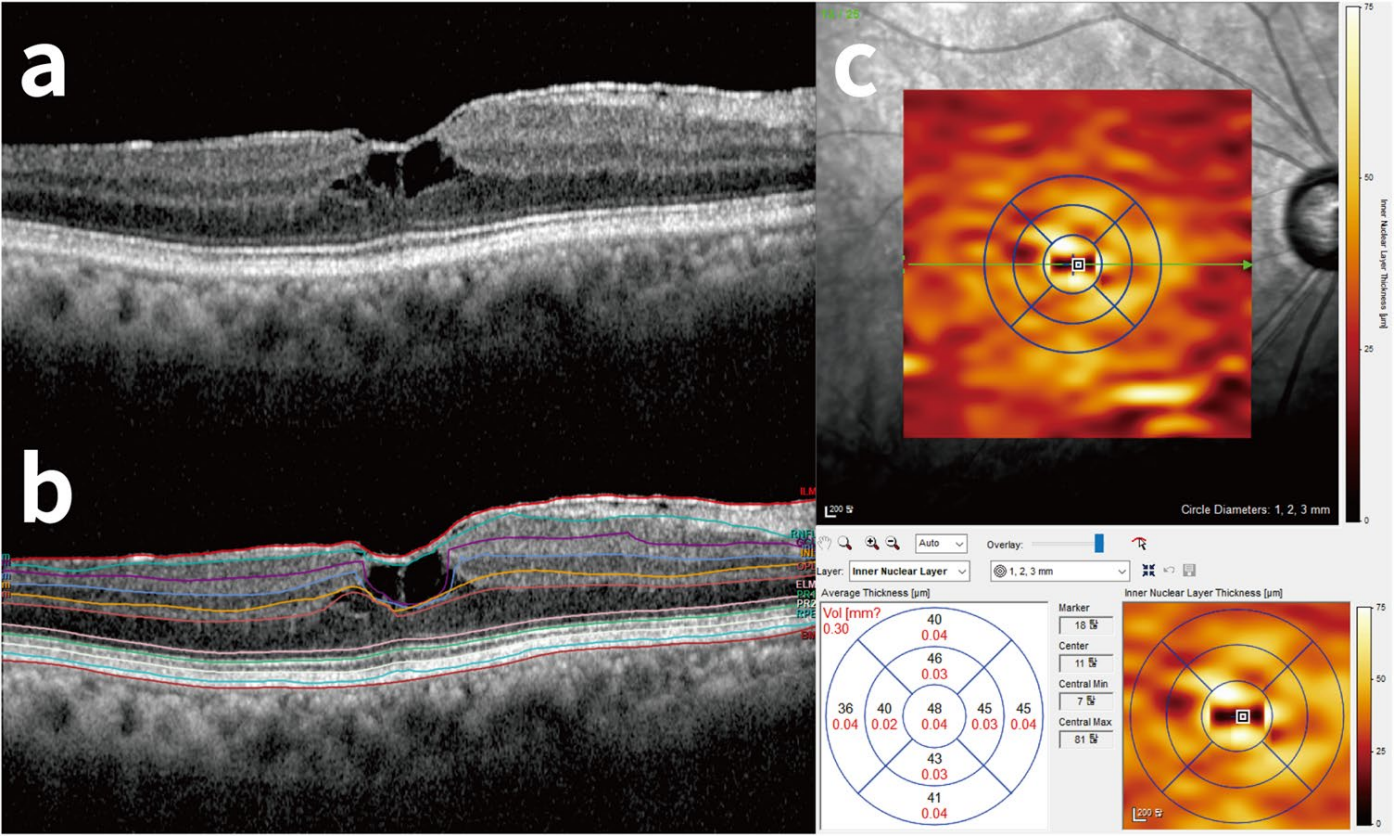
An example of automated retinal layer segmentation with spectral domain-OCT (Spectralis HRA + OCT; Heidelberg Engineering, Heidelberg, Germany). (**a**) A representative case of preoperative cystoid space. (**b**) Automated segmentation of retinal layers was performed by the built-in software (Heidelberg Eye Explorer, version 1.10.2.0; Heidelberg Engineering, Heidelberg, Germany) and manual correction was done afterwards when needed. (**c**) After segmentation, total retinal thickness of central 1 mm diameter region was analyzed. The thickness of nasal inner nuclear layer between central circle and inner ring (the area between 1 and 2 mm diameter circle) was analyzed on preoperative optical coherence tomography (OCT) image and postoperative OCT images.

A macular volume scan (20° × 20°) consisting of 25-line B-scan on spectral domain-OCT (Spectralis HRA + OCT; Heidelberg Engineering, Heidelberg, Germany) was performed before surgery and on each follow-up. The last available imaging data before surgery and all imaging data after the surgery were included in analysis. Automated segmentation of retinal layers was performed by the built-in software (Heidelberg Eye Explorer, version 1.10.2.0, Heidelberg Engineering, Heidelberg, Germany), and was manually corrected if necessary (Fig. 4a,b). Segmentation analysis was performed by two independent ophthalmologists (HYP, HSP) and the final confirmation was made by a retinal specialist (JYY, HSK). Analysis was performed within a 3-mm diameter circle centered on fovea. Diameters of central circle, inner ring, and outer ring were 1 mm, 2 mm, and 3 mm, respectively (Fig. 4c). Central macular thickness was measured manually on an OCT B-scan image consisting of fovea with embedded caliper system and was defined as distance between inner surface of retina and inner surface of retinal pigment epithelium at central fovea. Average foveal thickness was measured at central 1 mm diameter region of the grid. Thickness of nasal INL was defined as measured value between the central circle and the inner ring of the grid. Presence of IS/OS (inner and outer segment) disruption was evaluated. Preoperative cystoid space was defined as presence of any cystoid empty space detected in inner retinal layer, below ERM in any of OCT sections as shown in Fig. 4a, suggesting parenchymal loss of inner retinal layer. Distance between fovea and optic disc was measured manually on horizontal B-scan by embedded caliper system in OCT.

### Progressive nasal INL thickening

Progressive nasal INL thickening was defined as a time point at which 1.5-fold increase in the thickness of nasal INL after ERM surgery was shown. If nasal INL thickening was present at the last follow-up, OCT data on each visit were analyzed to identify the onset of the event.

### Statistical analysis

Student’s t-test was performed to compare continuous variables and Chi-square test was performed to compare categorical variables between nasal INL thickening group and non-nasal INL thickening group. Kaplan–Meier survival analysis and log-rank test were done to compare cumulative risk ratio of progressive nasal INL thickening between groups stratified by several categorical variables. Where nasal INL thickening shows up for the first time was regarded as an end point in the survival analysis. Follow-up ended when patients without INL thickening were censored. Univariate and multivariate logistic regression analysis were used to identify factors associated with progressive nasal INL thickening. Independent variables yielding *p*-values < 0.20 in univariate model were included in multivariate model. A *p*-value < 0.05 was considered statistically significant. Results are presented as mean ± standard deviation. All statistical analyses were performed with SPSS software (version 20; SPSS Inc. Chicago, IL, USA).

### Ethics approval

Institutional review board (IRB) approval was obtained (Siloam Eye Hospital IRB No. 2021-1).

### Consent to participate

Informed consent was waived after institutional review board (IRB) approval.

## Abbreviations

BCVA: Best corrected visual acuity
CI: Confident interval
ERM: Epiretinal membrane
ETDRS: Early Treatment Diabetic Retinopathy Study
ILM: Internal limiting membrane
INL: Inner nuclear layer
logMAR: Logarithm of the minimum angle of resolution
OCT: Optical coherence tomography
OR: Odds ratio
